# The Reliability and Acceptability of RDx-Based Tele-Controlled Subjective Refraction Compared with Traditional Subjective Refraction

**DOI:** 10.1167/tvst.11.11.16

**Published:** 2022-11-17

**Authors:** Jie Huang, Xiaoning Li, Tao Yan, Longbo Wen, Lun Pan, Zhikuan Yang

**Affiliations:** 1 Aier School of Ophthalmology, Central South University, Changsha, China; 2 Hunan Province Optometry Engineering Technology Research Center, Changsha, China; 3 Aier School of Optometry and Vision Science, Hubei University of Science and Technology, Xianning, China; 4 Nanchang Aier Eye Hospital, Nanchang, China; 5 Changsha Aier Eye Hospital, Changsha, China

## Abstract

**Purpose:**

The purpose of this study was to compare the reliability and acceptability of tele-controlled subjective refraction supported by RDx, a new technique that involves optical software designed for controlling phoropters remotely, to traditional subjective refraction.

**Methods:**

Sixty-five participants underwent tele-controlled subjective refraction and traditional subjective refraction randomly and nine of them underwent the second tele-controlled subjective refraction measurement on the same day. After their examinations, we distributed a validated satisfaction questionnaire to each participant. The elapsed time taken for refraction, sphere (S), cylinder (C), spherical equivalent (SE), vertical and oblique cylindrical vectors (J_0_ and J_45_), and best corrected visual acuity (BCVA) were compared. Age and refractive error type were included as covariates. Bland-Altman analysis was used to assess the agreement between both methods of refraction.

**Results:**

The mean age was 20.5 ± 5.9 years for all participants (aged 9 to 40 years); 57% were female participants. The repeatability analysis of tele-controlled method showed no significant differences for all parameters (*P* > 0.05). We found no statistical differences (*P* > 0.05) between tele-controlled subjective refraction and traditional subjective refraction for all parameters in either group. The mean difference and 95% limits of agreement for SE, J_0_, and J_45_ were −0.03 ± 0.36 diopters (D), −0.00 ± 0.57 D, and −0.01 ± 0.79 D, respectively. The tele-controlled method took more time to perform than the traditional one (*P* < 0.05). Completed questionnaires were returned by 55 participants (85%), and they showed high satisfaction and acceptance of the tele-controlled method (98%).

**Conclusions:**

Tele-controlled subjective refraction results agreed with traditional subjective refraction for all refraction components except for cylinder vectors. In addition, the broad acceptability of tele-controlled subjective refraction means practicability in clinical practice.

**Translation Relevance:**

The RDx-based tele-controlled method can provide an alternative to subjective refraction, especially in areas that lack experienced optometrists.

## Introduction

Uncorrected refractive error is recognized as the most common cause of preventable blindness and the leading cause of blindness in one-fifth of the global population.^1^ Objective refraction is widely used to detect refractive error due to its rapidness and maneuverability.^2^^–^^4^ Nevertheless, subjective refraction is considered to be the method of choice as it focuses on binocular vision and usually enables patients to obtain clear and comfortable vision afterward.^5^^–^^7^ However, this technique requires the expertise of an experienced optometrist in order to obtain a suitable spectacle prescription, and there is a significant shortage of optometrists and optometry resources in middle and low-income countries^8^ and rural areas.^9^

Faced with these challenges, novel technologies, such as telemedicine, have arisen that can expand the population served in certain areas and potentially improve delivery efficiency of overall health care.^10^^–^^12^ One software application in particular, RDx (Topcon, Tokyo, Japan), is designed to perform tele-controlled subjective refraction. Unlike traditional subjective refraction, the tele-controlled method enables optometrists to control phoropters remotely from anywhere. Because the tele-controlled method combines cloud technology with subjective refraction, follow-up visits can also be facilitated by accurately saving the refractive results to a cloud database and establishing personal refractive archives.

To evaluate the reliability and acceptability of RDx, in this study, we compared the consistency of refractive results and the elapsed time between RDx-supported tele-controlled subjective refraction and traditional subjective refraction. In addition, we also designed a questionnaire that was filled in after the participants’ examinations in order to investigate the participants’ attitudes toward tele-controlled subjective refraction.

## Methods

### Study Populations

The Research Ethics Committee of Nanchang Aier Eye Hospital (Nanchang, China) approved the study (No. 2020(018)), and all procedures in this study adhered to the tenets of the Declaration of Helsinki. All of the participants were informed of the study purpose and provided written informed consent. If participants were under the age of 16 years, we also obtained a signed written informed consent from their parents, guardians, or caregivers. To find participants for the study, we recruited patients who could cooperate during the examination and who had a best-corrected visual acuity of 0.3 logMAR units (20/40) or better from the optometry clinic of Nanchang Aier Eye Hospital. Patients were excluded if they had a history of ocular trauma or surgery, had rigid contact lenses currently in use, or had severe ocular disease that could interfere with the measurements.

### Examination Protocol

Initially, we evaluated the anterior ocular segments of a total of 130 eyes of 65 subjects using slit-lamp assisted microscopy and by taking a simple ocular history. Then, autorefraction was performed 3 times by an optometry assistant using a KR800 autorefractometer (Topcon, Tokyo, Japan), and the average value from this autorefraction was used as the start point of clinical subjective refraction. After this, both traditional and tele-controlled methods of subjective refraction were randomly performed by a trained and experienced optometrist under the same illumination with at least a 60-minute interval on the same day to minimize external influences on the measurements and results. The interval was put in place and the optometrist was asked not to see the same patient twice in a row in order to avoid potential bias from the optometrist's memory of previous subjective refraction. The sequence of subjective refractions was also performed randomly to avoid bias, and all refractions were performed without cycloplegia. Additionally, a second measurement of tele-controlled subjective refraction was scheduled at the same day after the first 2 measurements to access the repeatability of tele-controlled method, and 18 eyes of 9 subjects completed the repeated measurements of tele-controlled subjective refraction. Finally, all participants were invited to fill in our questionnaire after the ocular examination was completed.

### Traditional Subjective Refraction

As mentioned above, the refraction process in this study began with autorefraction. After that, spherical fogging, monocular maximum plus to maximum visual acuity (MPMVA), astigmatic correction with Jackson cross-cylinders, binocular balance, and binocular MPMVA were performed, in order. The end point was to consider the maximum plus sphere and minimum minus cylindrical power that provided the best visual acuity and comfortable vision for each patient. For the fogging technique, a positive lens of about +1.00 diopters (D) was added to fog the patient to a visual acuity of 20/60. All steps are in strict conformity with the principle of routine distance subjective refraction with a phoropter, trying to minimize the effect caused by accommodation. All measurements (spherical, cylindrical, axis, and best corrected visual acuity) were recorded. The binocular best corrected visual acuity (BBCVA) was assessed with trial spectacles. A Snellen chart displayed in the high-contrast (100%) digital screen at a 3-meter testing distance was used in both methods of refraction. The elapsed time in refractions was measured with a timer, and both in the traditional and tele-controlled methods, the time was measured from spherical fogging to final binocular MPMVA after seat height and pupillary distance adjustments were completed.

### Tele-Controlled Subjective Refraction

All procedures throughout tele-controlled refraction were based on RDx (a software application designed to perform tele-controlled subjective refraction) that had been installed in the optometry-clinic-side computer, and the remote optometrist was able to communicate with the selected patient and control the phoropter and LCD visual acuity chart on the store side, using a personal computer with RDx installed. An extranet connection was necessary to facilitate information synchronization between the two sides.

**Figure 1.**
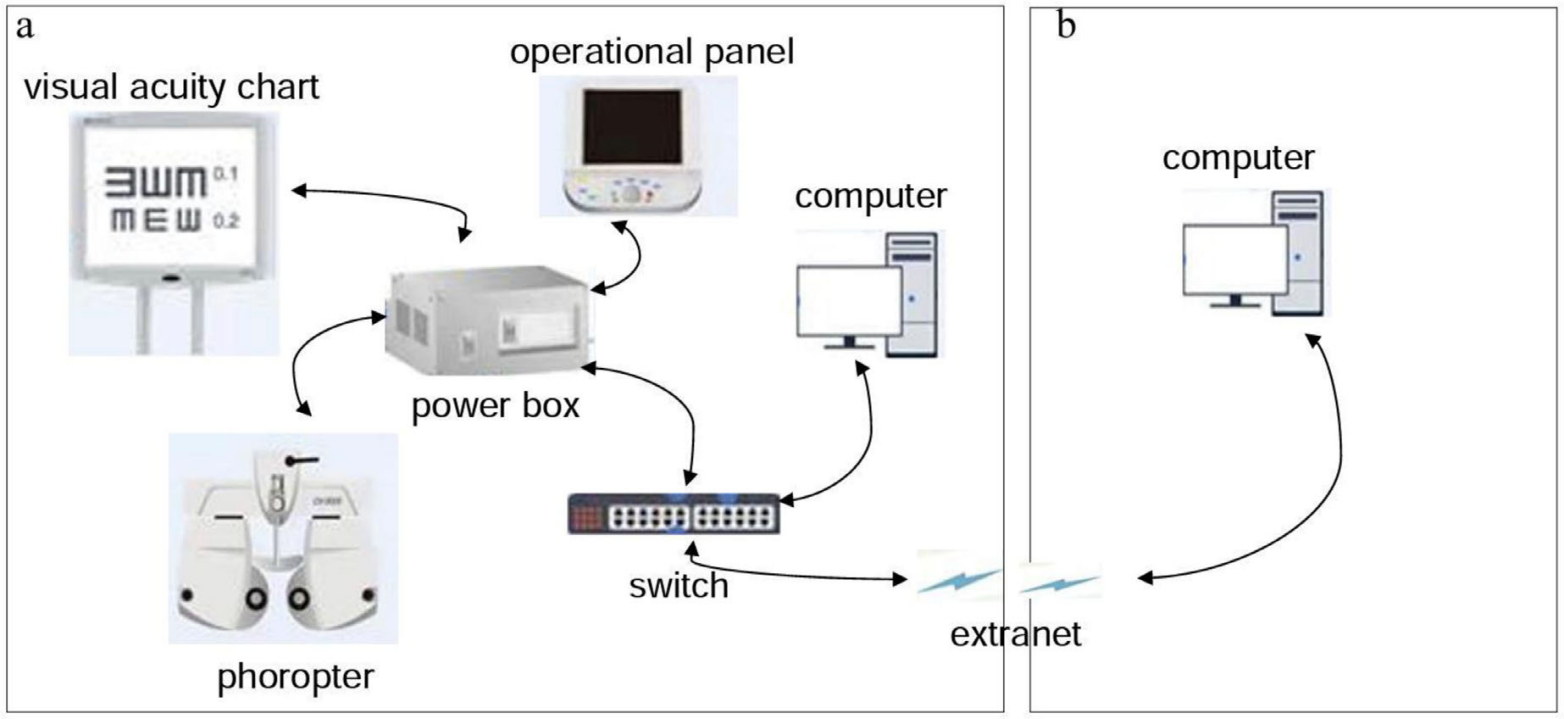
The specific schematic of the relationship between the main apparatuses. (**A**) The optometry-clinic-side and (**B**) the remote optometrist side.

The specific schematic of the above-described set up is shown in Figure 1, where the connection lines between the black arrows display the relationship among different devices. At the beginning of the tele-controlled refraction, the clinic side needed to add patients to the Patient Board section in the user interface of RDx and send requests to the remote optometrist. On the optometrist side, the remote optometrist received a list of patients waiting for examination on the RDx Dashboard. Subsequently, the optometrist just needed to double click to connect to one patient in the queue. Once both sides were connected to the session, the remote optometrist could launch a phoropter examination and begin tele-controlled subjective refraction. The results of the autorefraction and pretest data of the examinee also could be loaded by selecting the Fetch Pretest Data icon in RDx. The remaining steps of subjective refraction were the same as traditional subjective refraction, and all measurements and the elapsed time were recorded.

### Questionnaire Design

We initially established the first draft of the questionnaire through literature review and theoretical analysis. Then, we invited six optometrists, one intermediate statistician, and one intermediate assistant researcher to make suggestions. Finally, before administering the questionnaire to the patients in this study, we conducted a pilot test in which some optometrists were invited to complete the pilot questionnaire after undergoing tele-controlled subjective refraction. Feedback from both the advisory group and the optometrists was then used as a reference for questionnaire modification in order to make it as pertinent and comprehensive as possible. We categorized a questionnaire that we composed and that consisted of nine items into four sections: acceptability, satisfaction, communication, and trust in subjective refraction results. Respondents were required to respond according to their subjective feeling indicating to what extent they either agreed or disagreed with the item using a 7-point Likert response scale^13^ where 6 represented strongly agree, and 0 represented strongly disagree. A score of 0 to 2 was classified as disagreement with the statement, a score of 3 was classified as neither agreement nor disagreement, and a score of 4 to 6 was classified as agreement. We classified the questionnaire scores in the same manner as Doyle et al.^14^

### Statistical Analysis

Both eyes for each participant were included in our data analysis. We chose *P* values of less than 0.05 to indicate statistically significant test results and performed all statistical analysis using SPSS Statistics 26 (IBM, Chicago, IL). In addition, we converted all visual acuity measurements to the logarithm of the minimum angle of resolution units for statistical comparisons. The refractive results, such as spherical equivalent (SE), the vertical Jackson-Cross-Cylinder (J_0_), and the oblique Jackson-Cross-Cylinder (J_45_), were calculated through the following formulas^15^:

(1) SE=Sphere+1/2×Cylinder

(2) $$J_{0}=\left(-\mathrm{Cylinder}/2\right)\times \cos\left(2\times \alpha \right)$$

(3) $$J_{45}=\left(-\mathrm{Cylinder}/2\right)\times \sin\left(2\times \alpha \right)$$

To assess the normality of variables, we relied on the Shapiro-Wilk test, and we chose paired samples *t*-tests in the case of normal distributions and the Wilcoxon signed-rank test in the case of non-normal distributions. The distribution of refractive error in all samples was not normal, but the time of subjective refraction was. Hence, we expressed refractive measurements in the median and interquartile range for central tendency measures and dispersion and expressed elapsed time in mean ± standard deviation. In addition, Bland-Altman plots were constructed to visualize and assess the agreement between both methods of refraction. We also further subdivided the sample according to age and refractive error type, and made comparisons within each group.

Assuming an effect size of 0.5, our sample size calculations (G*Power3.1) indicate that 54 participants are sufficient for a power of 95% with a 2-sided paired *t*-test, adopting a significance value of 5%.

## Results

In a total of 65 participants with an age range of 9 to 40 years old (28 male and 37 female participants), averaging 20.5 ± 5.9 years were included in this study, and 37% of the subjects were under the age of 18 years. Demographic characteristics among subgroups can be found in Table 1.

**Table 1.** Demographic Characteristics of the Participants in the Research

Groups	*N* (%)	Age (Years)	Range (Years/Diopter)	Male (%)
Age				
Young children	12 (9)	11 ± 1.21	≥9 and ≤12	33
Teen	34 (26)	15.59 ± 0.93	≥13 and ≤17	53
Adult	84 (65)	23.79 ± 4.47	≥18	40
Refractive error type				
Mild hyperopia and emmetropia	10 (8)	20.6 ± 1.61	>−0.50 and <+2.00	20
Mild myopia	40 (31)	18.48 ± 6.81	≤−0.50 and >−3.00	40
Moderate and high myopia	80 (61)	21.44 ± 5.32	≤−3.00	48
Overall	130 (100)	20.46 ± 5.92	≥9 and ≤40 years	43

*N*, number of eyes.

### Repeatability Analysis

The test-retest differences of tele-controlled method were calculated and the results showed no statistically significant differences (*P* > 0.05) for any parameters. The mean difference ± 95% limits of agreement among the tests for SE, J_0_, and J_45_ were 0.03 ± 0.30 D, 0.11 ± 0.59 D, and 0.14 ± 1.17 D, respectively. The variation of SE measured by tele-controlled subjective refraction was small, whereas the results of oblique cylindrical vector showed great variability.

### Difference and Agreement Analysis

Table 2 summarizes the measures obtained from traditional subjective refraction and tele-controlled subjective refraction and the statistical comparison between the two methods. Compared to the traditional method, the tele-controlled method did not show statistically significant differences (*P* > 0.05) for any parameters. For sphere (S), SE, and cylinder (C) in the total group, the mean differences (traditional subjective refraction – tele-controlled subjective refraction) and their 95% limits of agreement were −0.03 ± 0.42 D, −0.03 ± 0.36 D, and 0.00 ± 0.39 D, respectively. However, even though there were no significant differences between the 2 methods in any measurements, we found wider ranges of the 95% limits of agreement for both J_0_ (±0.57 D) and J_45_ (±0.79 D). We also analyzed the percentage of refractive parameter differences between the 2 methods that fell within ±0.25 and ±0.50 D (see Table 2). We observed differences within ±0.25 D in 94% of eyes for SE, in 77% of eyes for J_0_ and in 72% of eyes for J_45_. As shown in Table 2, differences in all parameters between the 2 methods within ±0.50 D were ≥84% in all cases.

**Table 2.** Comparison Between Tele-Controlled and Traditional Subjective Refraction in the Total Group

	Traditional Method	Tele-Method				% Eyes	% Eyes
Parameters	Median (P_25_, P_75_)	Median (P_25_, P_75_)	*P*	MD (95% LoA)	Difference Mean (SD)	(± 0.25 D)	(± 0.50 D)
S	−3.25 (−4.75, −1.50)	−3.25 (−4.75, −1.50)	0.054	−0.03 (−0.45, +0.38)	−0.04 (0.21)	94	99
C	−0.50 (−1.00, −0.25)	−0.50 (−1.00, −0.25)	0.67	−0.03 (−0.39, +0.33)	0.01 (0.20)	94	99
SE	−3.63 (−5.03, −1.72)	−3.50 (−5.00, −1.59)	0.07	0.00 (−0.38, +0.40)	−0.03 (0.19)	94	99
J_0_	−0.07 (−0.18, 0.00)	−0.07 (−0.18,0.00)	0.95	0.00 (−0.58, +0.56)	0.00 (0.29)	77	89
J_45_	0.00 (−0.16, 0.24)	0.00 (−0.14,0.23)	0.72	−0.01 (−0.80, 0.77)	−0.01 (0.40)	72	84
BBCVA	−0.03 (0.05)	−0.02 (0.05)	0.09	/	−0.01 (0.02)	/	/

Refractive parameters data are expressed as median (*P_25_* and *P_75_*) and visual acuity data are expressed as mean (SD). S, sphere; C, cylinder; SE, spherical equivalent; J_0_, the Jackson cross cylindrical values at 180 degrees and 90 degrees; J_45_, the Jackson cross cylindrical values at 135 degrees and 45 degrees; BBCVA, binocular best corrected visual acuity; SD, standard deviation; MD, mean difference; LoA, limits of agreement in Bland-Altman Plots; D, diopter.

**Figure 2.**
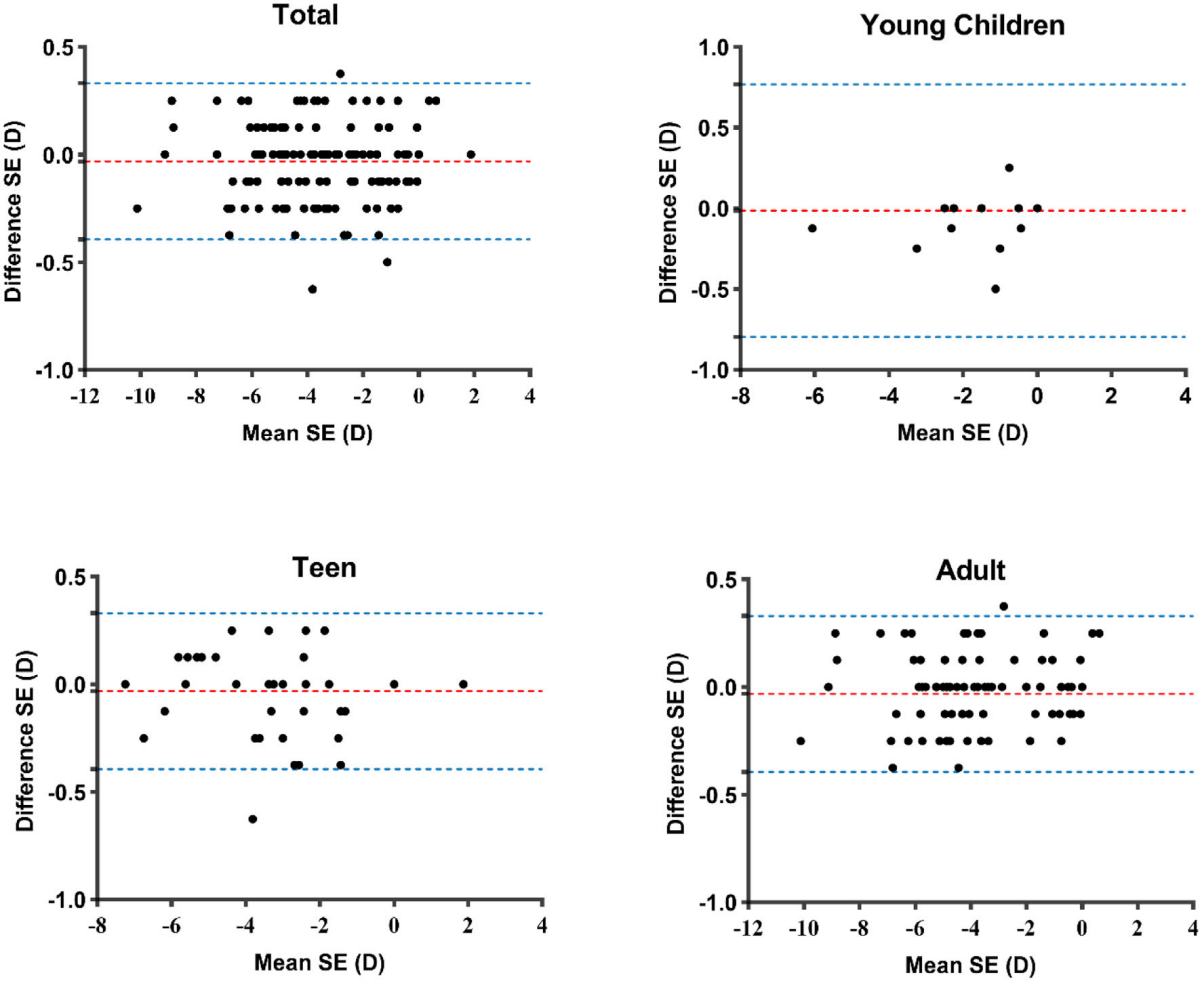
Bland-Altman plots between traditional and tele-controlled subjective refraction for total and different age groups. The *red lines* indicate the mean difference between the two methods and the *blue lines* show the 95% limits of agreement.

**Figure 3.**
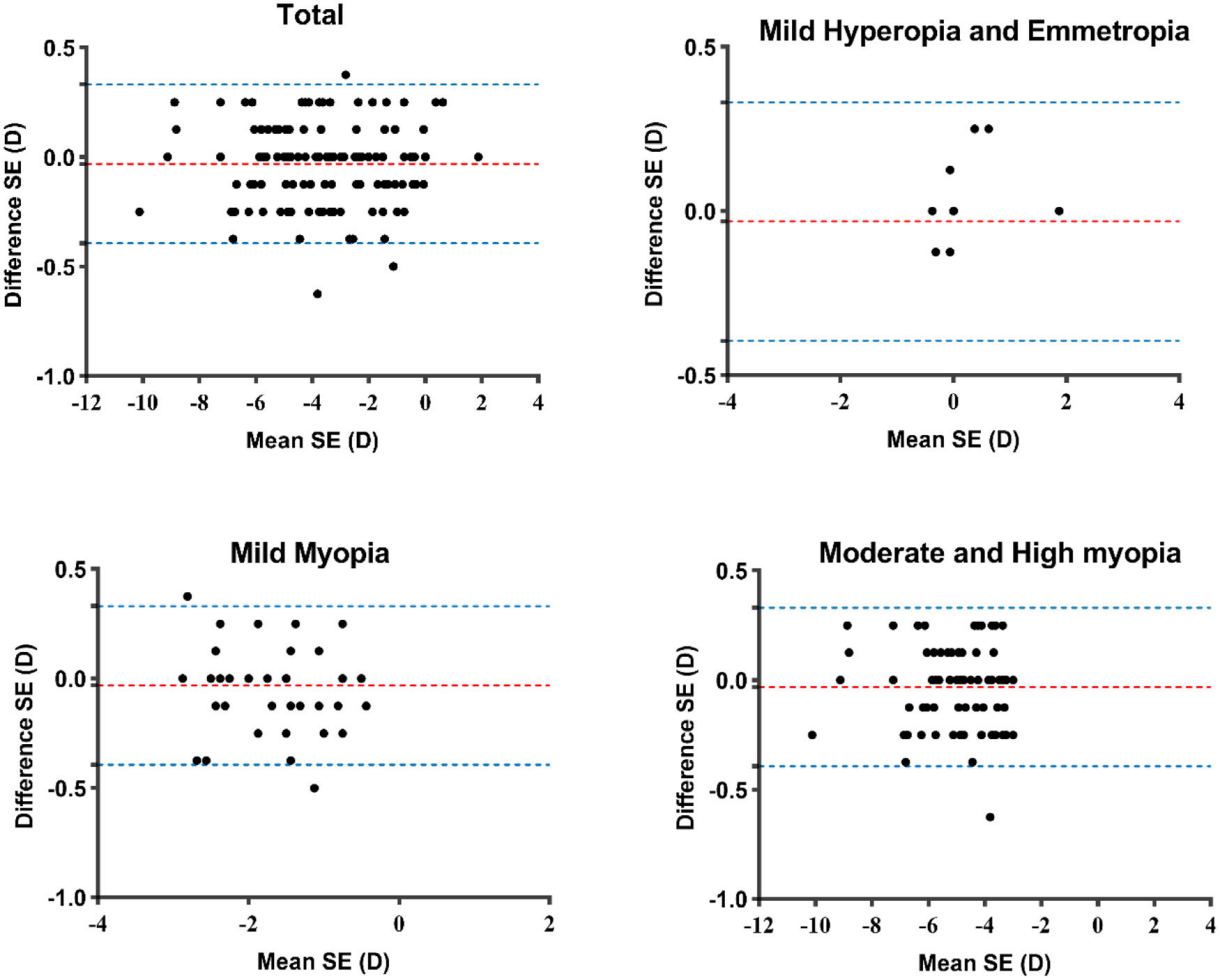
Bland-Altman plots between traditional and tele-controlled subjective refraction for total and different refractive error groups. The *red lines* indicate the mean difference between the two methods and the *blue lines* show the 95% limits of agreement.

Figure 2 shows the Bland-Altman plots comparing the SE measured with traditional and tele-controlled methods for the total, young children, teen, and adult groups. As shown in Figure 2, for SE, the 95% limits of agreement of young children, teens, and adults were ±0.37 D, ±0.41 D, and ±0.33 D, respectively. In addition, we encountered no significant differences (*P* > 0.05) for any parameters between eyes in the three age groups (Table 3). The agreement of SE measured with traditional and tele-controlled methods for the total and each refractive error group is shown in Figure 3 using Bland-Altman plots. Table 4 summarizes the statistical comparison and agreement between traditional and tele-controlled subjective refraction for SE, J_0_, and J_45_ in different refractive error groups. For SE, the mean difference and their 95% limits of agreement of mild hyperopia and emmetropia, mild myopia, and moderate and high myopia groups were 0.04 ± 0.26 D, −0.05 ± 0.38 D, and −0.04 ± 0.36 D, respectively. No significant differences (*P* > 0.05) were found between the two methods for any parameters in either refractive error group.

**Table 3.** Paired Comparison and Agreement Between Traditional and Tele-Controlled Subjective Refraction for SE, J_0_, and J_45_ in Different Age Groups

		Median (P_25_ and P_75_)				
Groups	Parameters	Traditional Method	Tele-Controlled Method	*P* Value	MD (95% LoA)	Difference Mean (SD)
Young children	SE	−1.44 (−2.47, −0.53)	−1.19 (−2.44, −0.59)	0.12	−0.09 (−0.46, 0.27)	−0.09 (0.19)
	J_0_	−0.09 (−0.29, 0.00)	0.00 (−0.11, 0.00)	0.09	−0.08 (−0.36, 0.21)	−0.08 (0.15)
	J_45_	−0.13 (−0.23, 0.00)	0.00 (−0.23, 0.17)	0.50	−0.05 (−0.75, 0.65)	−0.05 (0.36)
Teen	SE	−3.25 (−4.84, −2.13)	−3.25 (−4.97, −2.28)	0.15	−0.06 (−0.47, 0.35)	−0.06 (0.21)
	J_0_	0.00 (−0.08, 0.17)	−0.05 (0.15, 0.02)	0.28	0.06 (−0.42, 0.54)	0.06 (0.24)
	J_45_	0.00 (−0.11, 0.24)	−0.01 (−0.12, 0.16)	0.28	0.04 (−0.55, 0.63)	0.04 (0.30)
Adult	SE	−4.25 (−5.25, −2.00)	−4.25 (−5.25, −1.81)	0.32	−0.02 (−0.35, 0.31)	−0.02 (0.17)
	J_0_	−0.07 (−0.18, 0.00)	−0.10 (−0.21, 0.01)	0.98	0.00 (−0.63, 0.63)	0.00 (0.32)
	J_45_	0.00 (−0.16,0.21)	0.00 (−0.17,0.25)	0.45	−0.03 (−0.89,0.82)	−0.03 (0.44)

SE, spherical equivalent; J_0_, the Jackson cross cylindrical values at 180 degrees and 90 degrees; J_45_, the Jackson cross cylindrical values at 135 degrees and 45 degrees; MD, mean difference; LoA, limits of agreement in Bland-Altman Plots; SD, standard deviation.

**Table 4.** Paired Comparison and Agreement Between Traditional and Tele-Controlled Subjective Refraction for SE, J_0_, and J_45_ in Different Refractive Error Groups

		Median (P_25_ and P_75_)				
Groups	Parameters	Traditional Method	Tele-Controlled Method	*P* Value	MD (95% LoA)	Difference Mean (SD)
Mild hyperopia and emmetropia	SE	0.00 (−0.19, 0.56)	0.00 (−0.16, 0.31)	0.33	0.04 (−0.22, 0.30)	0.04 (0.13)
	J_0_	0.00 (−0.09, 0.03)	0.00 (−0.03, 0.08)	1	−0.03 (−0.45, 0.38)	−0.03 (0.21)
	J_45_	0.00 (−0.05, 0.05)	0.00 (−0.04, 0.00)	0.72	−0.01 (−0.14, 0.12)	−0.01 (0.07)
Mild myopia	SE	−1.56 (−2.34, −1.03)	−1.50 (−2.34, −0.88)	0.16	−0.05 (−0.42, 0.33)	−0.05 (0.19)
	J_0_	−0.02 (−0.11, 0.00)	0.00 (−0.09, 0.04)	0.18	−0.02 (−0.43, 0.39)	−0.02 (0.21)
	J_45_	0.00 (−0.15, 0.08)	0.00 (−0.12, 0.15)	0.39	−0.03 (−0.53, 0.47)	−0.03 (0.26)
Moderate and high myopia	SE	−4.88 (−5.84, −3.88)	−4.75 (−5.75, −3.78)	0.06	−0.04 (−0.40, 0.32)	−0.04 (0.18)
	J_0_	−0.07 (−0.21, 0.06)	−0.13 (−0.21, 0.00)	0.38	0.03 (−0.62, 0.68)	0.03 (0.33)
	J_45_	0.00 (−0.19, 0.24)	0.00 (−0.23,0.36)	0.93	−0.01 (−0.94, 0.93)	−0.01 (0.48)

SE, spherical equivalent; J_0_, the Jackson cross cylindrical values at 180 degrees and 90 degrees; J_45_, the Jackson cross cylindrical values at 135 degrees and 45 degrees; MD, mean difference; LoA, limits of agreement in Bland-Altman Plots; SD, standard deviation.

### Examination Efficiency Analysis

For elapsed time, the tele-controlled subjective refraction was statistically higher than the traditional method (*P* < 0.05). For the tele-controlled method, it took an average of 11.13 ± 2.02 minutes, and for the traditional subjective refraction it took an average of 10.45 ± 1.74 minutes. Furthermore, there were no statistical differences in elapsed time spent performing the two methods of subjective refraction between different age groups (10.79 ± 2.40 minutes vs. 11.40 ± 2.12 minutes vs. 11.07 ± 1.96 minutes; *P* = 0.80, least significant difference [LSD] *t*-test).

### Visual Acuity Analysis

The BBCVA was cumulatively at least 20/15 in 13.1% of eyes, and 20/20 in all eyes. In terms of the BBCVA results with both methods of refraction, they are summarized in Table 2. There were no significant differences between the two methods of refraction for the total group (*P* > 0.05). Additionally, we screened out participants with binocular cylinder power from −1.00 D to 0. Compared to the traditional method, the tele-controlled did not show statistically significant differences (−0.024(0.05) vs. −0.019(0.05), *P* = 0.18) in BBCVA of those participants.

### Questionnaire Analysis

Fifty-five questionnaires were completed (85%), and, among them, 38% were answered by male respondents. There was a wide range of respondents’ ages, ranging from 9 to 40 years old, with the largest proportion (56%) of respondents having ages 20 to 30 years old. We noted no significant differences in sex (*P* = 0.06, χ^2^ test) between participants who completed the questionnaire and those who did not complete the questionnaire, and respondents were older than those who did not fill out the questionnaire (21.29 ± 5.90 vs. 15.90 ± 3.84 years; *P* = 0.007, independent-samples *t*-test).

**Figure 4.**
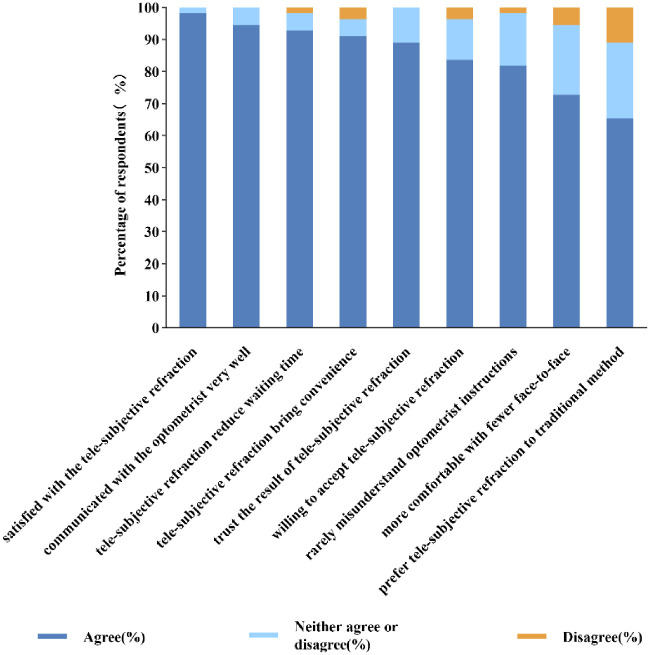
The percentage of respondents’ attitudes to each statement toward attitudes to the tele-controlled subjective refraction. Agreement with each statement is shown in *dark blue*, neither agreement nor disagreement in *light blue*, and disagreement in *yellow*.

Table 5 and Figure 4 provide details of responses relating to respondents’ attitudes toward tele-controlled subjective refraction. Over 90% of respondents answered that they were satisfied with tele-controlled subjective refraction, and approximately 5 in 6 respondents indicated that they would like to accept remote schema if possible. Finally, misunderstanding of the optometrists’ instructions was reported as infrequent (2%), and a good sense of trust in the results of the tele-controlled subjective refraction was illustrated by the fact that 89% of the respondents responded that they placed confidence in it.

**Table 5.** Participants’ Attitudes Toward Tele-Controlled Subjective Refraction

		Disagreement to Agreement (%)						
Statement		0	1	2	3	4	5	6
**Q1**	Overall, I was satisfied with the tele-subjective refraction.	0	0	0	1.8	7.3	52.7	38.2
**Q2**	I am willing to accept tele-subjective refraction in my daily life if possible.	0	0	3.6	12.7	7.3	36.4	40
**Q3**	I prefer tele-subjective refraction to traditional subjective refraction.	0	1.8	9.1	23.6	23.6	16.4	25.5
**Q4**	I think tele-subjective refraction can bring convenience to consultation process.	0	1.8	1.8	5.5	12.7	29.1	49.1
**Q5**	I think tele-subjective refraction can reduce the waiting time.	0	0	1.8	5.5	16.4	32.7	43.6
**Q6**	I feel more comfortable with fewer face-to-face communication during tele-subjective refraction.	0	0	5.5	21.8	16.4	32.7	23.6
**Q7**	I communicated with the optometrist very smoothly during tele-subjective refraction.	0	0	0	5.5	12.7	29.1	52.7
**Q8**	I rarely misunderstand the optometrist instructions during tele-subjective refraction.	0	0	1.8	16.4	12.7	20	49.1
**Q9**	I trust the result of tele-subjective refraction.	0	0	0	10.9	23.6	32.7	32.7

A score of 6 indicates that participants strongly agreed with the statement, and a score of 0 indicates that they strongly disagreed with the statement.

## Discussion

The purpose of this study was to explore the reliability and acceptability of tele-controlled subjective refraction. This was also the first time anyone has deeply investigated the participants’ attitudes toward tele-controlled subjective refraction. In our cross-sectional study, we found no statistically significant differences between traditional subjective refraction and tele-controlled subjective refraction for any parameters, and the 95% limits of agreement between the 2 methods of refraction in the S (±0.42 D), C (±0.39 D), and SE (±0.36 D) were both similar to a previous study on reproducibility of subjective refraction (±0.44 D^16^ for S, ±0.31 D^17^ for C, and ±0.37 D to ±0.56 D^18^^–^^21^ for SE). Researchers generally agree that the variation of refractive error should be at least ±0.50 D and consider this to be the minimum significant shift in a refractive state.^22^^,^^23^ Obviously, our results showed smaller 95% limits of agreement than the scientific minimum significant shift. It should, however, be noted that differences in the measurement of 0.5 D in clinical practice may lead to different diagnoses and treatment regimens by ophthalmologists. To better evaluate the practicality of tele-controlled subjective refraction, we established 0.25 D as a clinically meaningful difference between the 2 methods of refraction refractive error measurements as it is the smallest increment used for defining an eyeglass prescription. In this study, the percentage of the S, C, and SE differences between the 2 methods that fell within ±0.25 D were approximately 94% and considerably higher than a previous study.^24^ Therefore, we believe that this remote technology has the potential to help in clinical settings.

In terms of both vertical and oblique cylindrical vectors (J_0_ and J_45_), despite the results showing no statistically significant differences between the two methods (*P* = 0.95 and *P* = 0.72, respectively) and the mean differences appearing to be small, the lower degree of the agreement must be interpreted with caution. Considering the results of our Bland-Altman plot analysis, our study showed a little wider 95% limits of agreement for the J_0_ (±0.57 D vs. ±0.40 D^25^) and J_45_ (±0.78 D vs. ±0.43 D^26^) power vectors compared to a previous study that itself compared the repeatability of autorefraction. This difference may potentially be because autorefraction is good at avoiding the poor axial resolution of participants with cylinder power ≤1.00 D in the process of subjective refraction.^27^ In addition, previous research has suggested that the difference in axis has a negative correlation with the measured magnitude of the cylinder.^28^ Hence, 107 of 130 (82%) of the eyes included in our analysis belonged to the group with cylinder power ≤1.00 D, which may contribute to the wider 95% limits of agreement for J_0_ and J_45_.

Although our study showed a lower degree of agreement between J_0_ and J_45_ power vectors compared with other studies, the results may not be recognized as clinically meaningful. The differences in the J_0_ and J_45_ power vectors of more than ±0.50 D occurred in only approximately 20% of eyes. More importantly, all of the participants were able to achieve a BBCVA of 20/20 or better with the traditional method and with the tele-controlled method. This suggests that the difference in axis had no perceptible effect on the accuracy of the final prescriptions. Taking all these factors together suggests that tele-controlled subjective refraction has considerable value in clinical practice.

Tele-controlled refraction necessarily relies on good cooperation between optometrists and patients, and, for this study, we used patient age as a proxy for cooperation in our analysis. Our results show that no statistically significant differences (*P* ≥ 0.05) were present for any of the parameters in different age groups. Additionally, whether with the range of limits of agreement or the mean differences, all of the age groups showed similar values in the comparison between the two methods for SE, J_0_, and J_45_. Given the fact that we noted no statistical difference in examination efficiency between age groups and younger patients (the youngest was 9 years old), we considered that tele-controlled subjective refraction is applicable to most patients with good concentration and cooperation.

In relation to the examination efficiency, the elapsed time for the tele-controlled method was slightly longer than that for the traditional method, but could not be considered as having obvious clinical significance due to the lower difference (averaging 48 seconds longer). Because all the procedures were conducted online, we do note that the stability of the networks involved and the fluency of communication can affect the elapsed time for the tele-controlled method to a certain degree. Most notably, the longer time-consuming method may arise some restlessness of patients especially in younger children. However, those possible negative aspects may become a less of issue with the rapid development of communication technologies in the future.^29^

With regard to the acceptability of tele-controlled subjective refraction, although the tele-controlled method took longer than the traditional method, our questionnaire results showed that the majority of the subjects (98%) were satisfied with the tele-controlled method, and more than half of the respondents (66%) said that they preferred the tele-controlled method to the traditional method. Furthermore, patients’ positive attitudes toward the tele-controlled method in the majority of subjects could be due to the superior convenience it brings to the consultation process. In addition to this, the optometrist communicated with the subjects through a webcam and had fewer opportunities for face-to-face interaction, making tele-controlled refraction a better option for patients who may not want or be able to venture out during the ongoing coronavirus disease 2019 (COVID-19) pandemic.^30^ It is worth noting that all of the statements in the questionnaire are set in a positive direction and this may also be one of the reasons for the patients’ positive attitudes. Therefore, there is still a need for more comprehensive evidence to further clarify the patients’ attitude toward tele-controlled subjective refraction. For us, judging from the clinically acceptable results and positive attitudes, the tele-controlled method of refraction proved to be quite practical and therefore may become a valuable supplement for areas that lack experienced optometrists.

This study has several limitations. First, all subjective refraction was conducted without cycloplegia. As observed by Pei et al.,^17^ cycloplegic measurements are generally more positive or less negative than refractions without cycloplegia. Although enough fogging and standardized operations were performed to minimize the influences induced by accommodation,^31^ this may not be enough for individuals with hyperopia or under the age of 10 years.^32^ Hence, we urge caution and prudence when conducting tele-controlled subjective refraction on those patients. Second, all subjective refraction was conducted by one optometrist, resulting in lack of evaluation of inter-rater reliability. The potential variations between optometrists are worth to be explored in future studies. Besides, as only one optometrist was involved, the optometrists’ memory of the previous prescription may have introduced some bias. Although there may be variation in measurements caused by clinician bias, a single optometrist can at least in partly minimize this bias.^33^^,^^34^ However, the optometrist was required to follow end point judgment criteria strictly by striving to achieve the best visual acuity and most comfortable vision rather than merely going by participants’ previous prescriptions. Third, the sample size for repeatability analysis and subset analysis were relatively small. The low number of participants, especially in the young children’s group and the mild hyperopia and emmetropia group, might limit the statistical power. Additionally, it would be interesting and useful to explore if tele-controlled subjective refraction is reliable and acceptable in patients who are not familiar with traditional subjective refraction. Finally, we did not explore the variability of measures of tele-controlled subjective refraction in patients with ocular conditions. Further reliability analysis in patients with poor visual acuity and comprehensive interpretation of the viewpoints of optometrists may better serve clinical applications of tele-controlled subjective refraction.

## Conclusions

Our study shows that the refraction results of tele-controlled subjective refraction supported by RDx corresponds well with traditional subjective refraction except for cylinder vectors. However, the lower degree of agreement for the cylinder axis only has a minor effect on the accuracy of the final prescription. It is worth noting that the vast majority of people in our study had high satisfaction and acceptance of the tele-controlled method, although it takes a longer examination time. Tele-controlled subjective refraction not only may be a useful tool for areas that lack experienced optometrists but may also help to pave the way for more telemedicine services in the future.

## Data Availability

The data used to support the findings in this study are available from the corresponding author upon reasonable request.
